# Challenges in the diagnosis of ulcerative colitis with concomitant bacterial infections and chronic infectious colitis

**DOI:** 10.1371/journal.pone.0189377

**Published:** 2017-12-06

**Authors:** Wei-Chen Lin, Chen-Wang Chang, Ming-Jen Chen, Cheng-Hsin Chu, Shou-Chuan Shih, Tzu-Chi Hsu, Horng-Yang Wang

**Affiliations:** 1 Division of Gastroenterology, Department of Internal Medicine, Mackay Memorial Hospital, Taipei, Taiwan; 2 Division of Colon and Rectal Surgery, Mackay Memorial Hospital, Taipei, Taiwan; University Hospital Llandough, UNITED KINGDOM

## Abstract

Ulcerative colitis (UC) is a chronic inflammation of the large bowel characterized by diarrhea and a negative stool culture. However, several enteropathogens have been implicated as causative agents in UC. The differentiation between chronic infectious colitis (IC) and UC with concurrent infection is difficult owing to their similar clinical presentations. The study aimed to explore the presentations and diagnostic clues that enable differentiation between UC with concomitant infections and chronic IC. The study included 17 UC patients with a bacterial infection and 46 with chronic IC. The UC patients (47 ± 19 years) were younger than the chronic IC patients (58 ± 20 years) (P = 0.022). Bloody diarrhea was more common in UC than in chronic IC (58.8% vs 10.9%, P < 0.001). Previous antibiotic usage was a risk factor for chronic IC (5.9% vs 32.6%, P = 0.031). Malignancy was a common comorbidity of chronic IC (5.9% vs 34.8%, P = 0.022). UC patients had lower antibiotic response rates than chronic IC patients (60.0% vs 87.2%, P = 0.026). *Aeromonas* species and *Clostridium difficile* were common in both groups. Histological features of cryptitis and crypt abscess were useful in the diagnosis of UC (P = 0.052 and P = 0.016, respectively). Bloody diarrhea in a young adult, decreased response to antibiotic treatment, and results of endoscopy with biopsy are important features in the diagnosis of UC with bacterial infection.

## Introduction

Ulcerative colitis (UC), an inflammatory bowel disease (IBD) involving the large bowel, is characterized by the presence of diarrhea and bloody mucoid stool.[1] Exclusion of infectious etiology in the diagnosis of UC is important, as the symptoms of infectious colitis, as well as the endoscopic and histological findings, overlap with UC.[1,2] The prevailing theory of the pathogenesis of UC suggests that the intestinal immune system is inappropriately activated due to a confluence of genetic and environmental factors that leads to inflammatory tissue damage.[3] Imbalances in the gut flora or specific bacterial strains play an role in the inflammatory process of UC.[3]

A large epidemiological study showed that the hazard ratio of IBD was 2.4 (95% confidence interval [CI], 1.7–3.3) in a gastroenteritis cohort compared to a control cohort without a gastroenteritis episode.[4] A higher risk of UC was noted during the first year after the infective episode (hazard ratio, 3.7; 95% CI, 1.8–7.5). In this study, the most commonly identified bacteria causing the enteric infection was *Campylobacter* spp., followed by *Salmonella* spp. and *Shigella* spp.[4] Another study found concurrent enteral infections at the time of IBD diagnosis in 21% of cases.[5] *Shigella*, *Salmonella*, and *Yersinia* strains have been investigated as a possible cause of UC, and *Clostridium difficile* toxin has been associated with disease exacerbation.[6] The role of *Aeromonas* species in UC is controversial, but recent studies have suggested that it can trigger UC relapse.[7,8]

Chronic diarrhea is a common but challenging clinical scenario due to the presence of several hundred differential diagnoses.[9] Despite the use of invasive colonoscopy in chronic diarrhea, the majority of macroscopic findings are unremarkable.[10] The most common causes include irritable bowel syndrome, IBD, microscopic colitis, and malabsorption syndromes.[11] Infection is an uncommon cause of chronic diarrhea.[12] Bacteria are less likely and parasites are most likely to cause chronic infectious colitis (IC) in immunocompetent patients.[12] However, when diarrhea lasts more than 4 weeks, other differentiated diagnoses such as UC should be considered.

To date, a causative bacterial agent for UC has not been identified because of the complexity of the colonic ecosystem.[2] Some commensal bacteria might become pathogenic under certain circumstances, and most bacteria live within the lumen without entering the mucosa.[2] The differential diagnoses of UC and IC may be complicated by their clinical, endoscopic, and histologic features,[2] especially in cases of undiagnosed UC complicated by infection. Therefore, in the present study, we compared the manifestations and the diagnostic clues of chronic IC versus UC with a concomitant bacterial infection.

## Materials and methods

### Patient selection

Patients with UC and chronic diarrhea and a concomitant bacterial infection at Mackay Memorial Hospital, Taipei Medical Center between January 2014 and March 2017 were enrolled. The diagnosis of UC was based on a clinical evaluation of the patient’s medical history, clinical evaluation and typical endoscopic and histological findings. Chronic diarrhea is defined as an increased number of unformed stools (three or more times per day) compared with the normal state lasting at least 4 weeks.[11] Chronic infectious colitis is characterized by chronic diarrhea with a positive stool test (mucus, white blood cells, red blood cells, and culture for bacterial pathogens), and/or mucosal inflammation on colonoscopy. This study was approved by the Institutional Review Board of Mackay Memorial Hospital (reference number: 17MMHIS089), which waived the requirement for informed consent because of its retrospective design. Patient information was anonymized and de-identified prior to the analysis.

### Data collection

The database included computerized medical records of demographic data including age, sex, and underlying comorbidities. All stool samples were analyzed for parasites, neutral fat, occult blood, and pus cells. Stool samples were sent to a laboratory for routine culture (*Salmonella*, *Shigella*, *Campylobacter*, *Vibrio*, *Aeromonas*, *Plesiomonas*, and *C*. *difficile*). Due to the low sensitivity of *C*. *difficile* culture, a combined qualitative enzyme immunoassay of both toxin A/B and glutamate dehydrogenase was performed.

Clinical presentations included bloody diarrhea, fever (>38.0°C), weight lost and abdominal pain. Medical history included antibiotic, proton-pump inhibitor, nonsteroidal anti-inflammatory drug, and immunosuppressant drug use within the previous 6 weeks. Laboratory data included complete blood count, C-reactive protein (CRP), and albumin level. Histological features included cryptitis, crypt abscess, Paneth cell metaplasia, and plasma/lymphocyte infiltrates.

### Statistical analysis

Descriptive statistics for continuous variables are reported as mean ± standard deviation (SD). The categorical variables were described using frequency distributions and are reported as n (%). P values were based on a t-test for continuous variables and the chi-square or Fisher’s exact test was used for the categorical variables. The statistical analysis was performed using the STATA statistical package (version 12.0; Stata, College Station, TX, USA). Tests were two-tailed with a significance level of 0.05.

## Results

### Demographic features

The demographic and disease characteristics of patients with UC and chronic IC are summarized in Table 1. There were 17 UC patients with a concomitant bacterial infection and 46 patients with chronic IC. The mean age of the UC patients (46.5 ± 19.2 years) was lower than that of the chronic IC patients (57.9 ± 19.9 years) (P = 0.022). The male-to-female ratio of UC and chronic IC patients was 1.4:1 and 0.9:1, respectively (P = 0.438). Bloody diarrhea is more common in UC than in chronic IC (58.8% vs 10.9%, P < 0.001). Minor differences were seen in symptoms such as abdominal pain, fever, and weight loss.

**Table 1:** Baseline characteristics among UC and chronic IC patients.

	UC (N = 17)	IC (N = 46)	P value
Age	46.5±19.2	57.9±19.9	0.022^†^
Gender (male)	10(58.8)	22(47.8)	0.438^†^
**Symptoms**			
Bloody diarrhea	10(58.8)	5(10.9)	<0.001^†^
Abdominal pain	2(11.8)	6(13.0)	0.892^†^
Fever	2(11.8)	11(23.9)	0.290^†^
Weight lost	3(17.6)	12(26.1)	0.485^†^
**Medical history**			
Antibiotics use	1(5.9)	15(32.6)	0.031^†^
Proton pump inhibitor	4(23.5)	8(17.4)	0.582^†^
NSAID	4(23.5)	12(26.1)	0.836^†^
Immunosuppressants	3(17.6)	2(4.3)	0.083^†^
**Comobility**			
Liver cirrhosis	1(5.9)	6(13.0)	0.422^†^
Renal impairment	0(0)	7(15.2)	0.088^†^
Diabetes mellitus	1(5.9)	13(28.3)	0.058^†^
Malignancy	1(5.9)	16(34.8)	0.022^†^
**Laboratory**			
CRP (mg/dL)	4.9±5.2	6.6± 8.7	0.272^§^
Hemoglobin (g/L)	11.4± 2.4	11.3± 2.2	0.468^§^
Leukocyte (10^9^/L)	8.6± 4.3	7.7± 3.7	0.192^§^
Platelet (10^9^/L)	351.0± 179.5	225.1± 118.4	0.001^§^
Albumin (g/dL)	3.3±0.7	3.2±0.6	0.368^§^
**Management**			
Antibiotics	15(88.2)	39(84.8)	0.782^†^
5-ASA	15(88.2)	3(6.5)	<0.001^†^
Steroid	5(29.4)	3(6.5)	0.015^†^
Probiotics	2(11.8)	1(2.2)	0.113^†^
**Response to antibiotic treatment**			
	9/15(60.0)	34/39(87.2)	0.026^†^

Abbreviations: NSAID, nonsteroidal anti-inflammatory drugs; 5-ASA, 5-aminosalicylic acid

P value was determined using t-test^§^ or

Chi-squared test^†^.

In previous medical history, antibiotic use was a risk factor of chronic IC (5.9% vs 32.6%, P = 0.031). There were no differences in previous proton pump inhibitor, nonsteroidal anti-inflammatory drug, or immunosuppressant drug use. Malignancies were less common in UC than in chronic IC (5.9% vs 34.8%, P = 0.022). The mean platelet count (351.0 ± 179.5 vs 225.1 ± 118.4 10^9^/L, P = 0.001) was higher among UC patients than among chronic IC patients. The hemoglobin, leukocyte, albumin, and CRP levels were not significantly different. In terms of clinical management, 5-aminosalicylic acid (88.2% vs 6.5%, P < 0.001) and steroid (29.4% vs 6.5%, P = 0.015) were more commonly used in the UC group. Antibiotics were the most common first-line agent in both groups (differences not statistically significant). UC patients had a lower response rate to antibiotic treatment than chronic IC patients (60.0% vs 87.2%, P = 0.026).

### Infection in UC

Seventeen UC patients with a concomitant bacterial infection were enrolled; of them, eight were initially diagnosed (Table 2). Pancolitis was the most common disease site (52.9%), followed by left-side colitis (29.4%) and proctitis (17.6%). Two patients did not undergo colonoscopy due to the disease occurring during a UC flare-up. Among 11 of 15 (73.3%) patients with histologic UC features, six were initial diagnosis. Patients 1 and 2 presented with diarrhea that was refractory to antibiotic treatment and diagnosed 1 month after endoscopic follow-up. Patients 3 and 4, who had no histologic features, were diagnosed by their endoscopic features and good response to 5-aminosalicylic acid treatment. *Aeromonas* species (41.2%, 7/17) were the most common pathogen in UC patients with a concurrent infection, followed by *C*. *difficile* (29.4%, 5/17).

**Table 2:** Clinical characteristics of UC patients with a concomitant infection.

No	Age	Gender	Initial diagnosis	Involved area	UC histology	Antibiotic	UC Treatment	pathogen
1	21	M	Yes	E2	Yes	Yes	Nil	Aeromonas
2	41	M	Yes	E2	Yes	Yes	Nil	Campylobacter
3	34	M	Yes	E3	No	Yes	5-ASA	Aeromonas
4	44	F	Yes	E3	No	Yes	5-ASA	Aeromonas
5	22	F	Yes	E3	Yes	Yes	5-ASA	Campylobacter
6	11	F	Yes	E2	Yes	No	5-ASA	Aeromonas
7	23	M	Yes	E2	Yes	Yes	5-ASA	C. difficile
8	50	M	Yes	E3	Yes	Yes	5-ASA+ steroid	Aeromonas
9	56	F	No	E3	Yes	Yes	5-ASA	Plesiomonas
10	47	M	No	E2	Yes	Yes	5-ASA+ steroid	C. difficile
11	52	M	No	E3	Yes	Yes	5-ASA+ steroid	Aeromonas
12	60	F	No	E1	Yes	Yes	5-ASA+ steroid	Salmonella
13	69	F	No	E3	Yes	Yes	5-ASA	C. difficile
14	80	F	No	E3	Nil	Yes	5-ASA	C. difficile
15	36	M	No	E1	Nil	No	5-ASA	Aeromonas
16	81	F	No	E3	No	Yes	5-ASA+ steroid	C. difficile
17	63	M	No	E1	No	Yes	5-ASA	Plesiomonas, Campylobacter

Abbreviations: M, male; F, female; E1, proctitis; E2, left-sided UC; E3, extensive UC; 5-ASA, 5-aminosalicylic acid

### Histological features

Biopsies were performed at the time of infection in 15 of 17 patients with UC and 27 of 46 patients with chronic IC (Table 3). Cryptitis (13.3% vs 0%, P = 0.052) and crypt abscess (20% vs 0%, P = 0.016) were commonly seen in UC patients. There were no differences in plasma/lymphocyte infiltration (P = 0.344) or Paneth cell metaplasia (P = 0.174) in the two groups.

**Table 3:** Histological findings of UC and chronic IC cases.

	UC (N = 15)	IC (N = 27)	P value
Cryptitis	2 (13.3)	0(0)	0.052
Crypt abscess	3 (20.0)	0(0)	0.016
Paneth cell metaplasia	1(6.6)	0(0)	0.174
Plasma/lymphocyte infiltrate	6(40.0)	7(25.9)	0.344

### Pathogenicity of different species

*Aeromonas* species was the most common pathogen in 62.5% (5/8) patients with an initial UC diagnosis and 47.8% (22/46) of patients with chronic IC (S1 Table). *C*. *difficile* was the most common pathogen in 44.4% of UC patients with flare-ups and the second most common pathogen (30.4%, 14/46) in chronic IC patients. However, there was no statistically significant difference in pathogens between UC and chronic IC.

## Discussion

Diarrhea was long thought to be caused by infectious agents such as bacteria. In 1875, Wilks and Moxon first described UC as a separate entity from IC.[13] Thereafter, UC was suggested to be a chronic remitting-relapsing disease of the large colon for which an infective cause should be excluded.[1] Increasing evidence shows that a persistent infection with a specific pathogen was related to degradation of the luminal protective structures and led to UC.[2] In the present retrospective study, we observed 17 UC patients with a concomitant bacterial infection and compared them to a control group of 46 patients with chronic IC. *Aeromonas* species and *Clostridium difficile* were common causative pathogens in UC and chronic IC. UC patients tended to be younger. Symptoms of bloody diarrhea, poor response to antibiotics treatment and histological UC features are useful in the initial diagnosis of UC with a concomitant infection.

Bloody diarrhea is the most common characteristic symptom of UC,[1] but elderly patients have a less typical presentation.[14] In a prospective study of cases of acute bloody diarrhea (<7 days) treated in emergency rooms caused by infectious agents, *Shigella* species was the most common pathogen (15.3%), followed by *Campylobacter* and *Salmonella* spp.[15] In our study, *Aeromonas* species are the most common pathogens in UC and chronic IC, followed by *C*. *difficile*, which are quite different from the pathogens in acute bloody diarrhea. In Taiwan, the most common etiologic agent in infectious diarrhea seen in community clinics is *Campylobacter* species, and *C*. *difficile* is the most common pathogen in children. There was no sex difference in the distribution of enteric pathogens in both adults and children. [16]

The most common symptom of *Aeromonas* infection is acute self-limiting diarrhea, while bloody diarrhea and chronic indolent diarrhea have also been reported.[17] *Aeromonas* can be a trigger of flare-ups or the development of IBD and cause a more severe infection in IBD patients than in non-IBD patients.[8] The incidence of *C*. *difficile* in IBD has doubled in recent years, increasing significantly in UC patients.[18] *C*. *difficile* can induce or mimic a UC flare-up, and patients with a concomitant *C*. *difficile* infection experience increased morbidity and mortality rates.[19]

A previous study showed that thrombocytosis is associated with surrogate markers for UC because the platelets are potent proinflammatory cells and inflammatory amplifiers in chronic inflammatory conditions.[20] The platelet count is markedly increased during the active stage of UC (defined as platelet count >450×10^9^/L), making it a rational marker for therapeutic intervention.[21] In this study, the mean platelet count was higher in the UC group, but remained within the upper limit of normal. This finding may be associated with chronicity in our patients, making the platelet count less useful for differential diagnosis. CRP levels increased with disease extent at UC diagnosis and could be used as a predictor of the need for surgery.[22] The levels of nearly 70% UC patients were within the normal range;[22] therefore, it was less useful as a marker for UC than for Crohn’s disease.

Histological features of chronicity such as a distorted crypt architecture, Paneth cell metaplasia, and increased plasma cell/lymphocyte infiltrates can help with the diagnosis of UC.[23] In this study, 73.3% (11/15) of UC patients have typical histology and crypt abscess was the strongest histological predictor of UC. The presence of plasma/lymphocyte infiltrates was not a useful marker since 25.9% (7/27) of chronic IC patients also had such features. A previous study showed that basal plasmacytosis was the most useful feature that differentiated UC from IC; however, 81% of those IC patients had acute-onset disease and did not show any histological UC features.[5] Another study revealed that chronic IC may produce a histological pattern of chronic active colitis that mimics UC.[24] The histological features are often not present during the initial phase of UC.[25] Crypt distortion and basal plasmacytosis first appear 2 weeks after infection and subsequently increase in frequency.[25] Therefore, the most suitable time to obtain a biopsy was 4–6 weeks after the first UC attack.

### Limitations

First, because bacterial infection is rarely a cause of chronic diarrhea and UC, the small sample size and retrospective design of this study might have led to patient selection bias. A prospective, large-scale study is needed to determine the role of enteric pathogens in UC. Second, 41.3% (19/46) of the clinical responses of chronic IC patients to antibiotic treatment were not confirmed with follow-up endoscopy, indicating that the possibility of UC could not be completely excluded. A short course of antibiotic therapy proved effective in patients with any stage of UC.[26] Third, it is very difficult to determine whether UC is caused by bacterial colonization or a new infection in a newly diagnosed patient. We cannot exclude the possibility that other agents are responsible for UC owing to the complexity of the colonic ecosystem. Most bacteria live within the lumen and do not enter the mucosa, so microbiological investigations of rectal biopsy samples may provide more solid evidence.

## Conclusions

Making the initial diagnosis of UC in patients with concomitant bacterial infections and diffuse continued mucosal inflammation in the colonoscopy is challenging. The combination of bloody diarrhea in a young adult and poor response to antibiotic treatment is an important clue to the possibility of undiagnosed UC. Follow-up colonoscopy with a biopsy wound help provide solid evidence for UC diagnosis.

## Supporting information

S1 Table Pathogens identified in cases of initially diagnosed UC, UC flare-up, and chronic IC. (DOCX)
