# Supplementary material for: Challenges in the diagnosis of ulcerative colitis with concomitant bacterial infections and chronic infectious colitis
Source: PLoS One. 2017 Dec 6;12(12):e0189377. doi: 10.1371/journal.pone.0189377 (PMC5718429; doi:10.1371/journal.pone.0189377)
Supplement: S1 Table — (DOCX) [file pone.0189377.s001.docx]

**S1 Table.** Pathogens identified in cases of initially diagnosed UC, UC flare-up, and chronic IC

| **Pathogens** | **UC (n=8)** | **UC flare up(n=9)** | **IC (n=46)** | **P value** |
| --- | --- | --- | --- | --- |
| Aeromonus | 5(62.5) | 2(22.2) | 22(47.8) | 0.315 |
| Clostridium difficile | 1(12.5) | 4(44.4) | 14(30.4) | 0.482 |
| Campylobacter | 2(25.0) | 1(11.1) | 7(15.2) | 0.740 |
| Plesiomonas | 0(0) | 2(22.2) | 2(4.3) | 0.062 |
| Shigella | 0(0) | 0(0) | 1(2.2) | 0.841 |
| Salmonella | 0(0) | 1(11.1) | 2(4.3) | 0.476 |
